# Epigenetic Programming Through Breast Milk and Its Impact on Milk-Siblings Mating

**DOI:** 10.3389/fgene.2020.569232

**Published:** 2020-10-02

**Authors:** Hasan Ozkan, Funda Tuzun, Serpil Taheri, Peyda Korhan, Pınar Akokay, Osman Yılmaz, Nuray Duman, Erdener Özer, Esra Tufan, Abdullah Kumral, Yusuf Özkul

**Affiliations:** ^1^Department of Pediatrics, Division of Neonatology, Faculty of Medicine, Dokuz Eylul University, İzmir, Turkey; ^2^Department of Medical Biology, Faculty of Medicine, Erciyes University, Kayseri, Turkey; ^3^Erciyes University’s Betul-Ziya Eren Genome and Stem Cell (Genkok), Kayseri, Turkey; ^4^İzmir Biomedicine and Genome Center, Dokuz Eylul University, İzmir, Turkey; ^5^Department of Medical Laboratory Techniques, İzmir Kavram University, İzmir, Turkey; ^6^The Experimental Animal Laboratory, Faculty of Medicine, Dokuz Eylul University, İzmir, Turkey; ^7^Department of Pathology, Faculty of Medicine, Dokuz Eylul University, İzmir, Turkey; ^8^Department of Medical Genetics, Faculty of Medicine, Erciyes University, Kayseri, Turkey

**Keywords:** breast milk, epigenetic regulatory mechanisms, miRNA, transgenerational inheritance, cross-fostering, life span

## Abstract

**Background:**

The epigenetic effects of transmission of certain regulatory molecules, such as miRNAs, through maternal milk on future generations, are still unknown and have not been fully understood yet. We hypothesized that breastfeeding regularly by adoptive-mother may cause transmission of miRNAs as epigenetic regulating factors to the infant, and the marriage of milk-siblings may cause various pathologies in the future generations.

**Results:**

A cross-fostering model using a/a and *A^*vy*^/a* mice had been established. F2 milk-sibling and F2 control groups were obtained from mating of milk-siblings or unrelated mice. Randomized selected animals in the both F2 groups were sacrificed for miRNA expression studies and the remainings were followed for phenotypic changes (coat color, obesity, hyperglycemia, liver pathology, and life span). The lifespan in the F2 milk-sibling group was shorter than the control group (387 vs 590 days, *p* = 0.011) and they were more obese during the aging period. Histopathological examination of liver tissues revealed abnormal findings in F2 milk-sibling group. In order to understand the epigenetic mechanisms leading to these phenotypic changes, we analyzed miRNA expression differences between offspring of milk-sibling and control matings and focused on the signaling pathways regulating lifespan and metabolism. Bioinformatic analysis demonstrated that differentially expressed miRNAs were associated with pathways regulating metabolism, survival, and cancer development such as the PI3K-Akt, ErbB, mTOR, and MAPK, insulin signaling pathways. We further analyzed the expression patterns of miR-186-5p, miR-141-3p, miR-345-5p, and miR-34c-5p and their candidate target genes Mapk8, Gsk3b, and Ppargc1a in ovarian and liver tissues.

**Conclusion:**

Our findings support for the first time that the factors modifying the epigenetic mechanisms may be transmitted by breast milk and these epigenetic interactions may be transferred transgenerationally. Results also suggested hereditary epigenetic effects of cross-fostering on future generations and the impact of mother-infant dyad on epigenetic programming.

## Background

As an epigenetic regulator, breast milk provides growth factors, immune factors, microbiota, stem cells, and microRNAs (miRNAs) (Kosaka et al., 2010; Hassiotou et al., 2012; Alsaweed et al., 2016; Melnik and Gerd, 2017). Lactation-specific miRNAs are secreted as extracellular vesicles (exosomes) derived from mammary gland epithelial cells, reach the systemic circulation of the newborn infant, mediates cellular communication between the mother and her nursing infant and then, may exert gene regulatory functions in the infant (Kosaka et al., 2010; Baier et al., 2014; Arntz et al., 2015; Alsaweed et al., 2016). The epigenetic effects of transmission of certain regulatory molecules, such as miRNAs, through maternal milk on future generations, are still unknown and have not been fully understood yet.

Currently, there are more than 2000 miRNAs that have been discovered in humans and it is believed that they collectively regulate one third of the genes in the genome. For instance, human milk provides abundant amounts of miRNA-148a, miR-152, miR-29b, and miR-21, which all target DNA methyl transferases (DNMTs) that potentially affect whole genome DNA methylation patterns leading to genome-wide DNA hypomethylation, and thus modifies gene expression (Bodo and Melnik, 2017; Melnik and Gerd, 2017). Continued uptake of milk-derived exosomes that carry DNMTs targeting miRNAs may promote diabesity, allergy, neurodegenerative diseases, and cancer later in life (Melnik and Schmitz, 2017).

The milk of each mammal is unique for its own offspring. However, due to various problems caused by the baby or mother, babies can not be fed with their own mother’s milk. In such cases, alternative breastfeeding practices allowing human milk sharing, such as cross−fostering or donor-milk banking are being considered (Arslanoglu et al., 2010; Palmquist and Doehler, 2016). In traditional cross-fostering, a wet nurse is a lactating woman who breastfeeds a child who is not her own; and individuals who are not biological siblings but breastfed by the same woman are defined as milk-siblings. To our knowledge, there is no study exist in the literature evaluating the issue of milk-siblingship and milk-sibling marriage on the basis of scientific evidence.

Until recently, transition of hereditary material only attributed to the Mendelian law. Therefore, it was impossible to understand how milk-sibling marriage could lead to heritable genetic transmission and increased risk of genetic diseases in future generations. Recent advances in epigenetic science have shown that there may be new and different perspectives. After the discovery of epigenetic regulatory factors, especially miRNAs, in breast milk, the milk-sibling hypothesis was first proposed in 2012 by our study group (Ozkan et al., 2012).

In this study, we hypothesized that breastfeeding regularly by adoptive -mother will cause transmission of some miRNAs as epigenetic regulating factors to the infant, and the marriage of milk-siblings may cause various pathologies in the future generations (Ozkan et al., 2012). Since it was impossible to create a model that will adapt our hypothesis to the human, an experimental model using a/a and *A^*vy*^/a* mice on C57Bl6J background had been established to test this hypothesis. To our knowledge, this is the first study evaluating the issue of cross-fostering, and milk-sibling mating on the basis of scientific evidence.

## Materials and Methods

### Animals

The study protocol was approved by the Dokuz Eylul University Medical Faculty, Animal Care and Use Committee. All experiments were performed in accordance with relevant guidelines and regulations. *A*^*vy*^/*a* and a/a mice on C57Bl6J background obtained from Missouri University, MMRRC laboratory via cryopreservation were used (RRID: MMRRC_000375-MU). All subjects were kept under standard conditions with a 12:12-h light-dark cycle and had free access to tap water and a standard pellet diet.

### Study Groups and Cross Fostering

**FIGURE 1:**
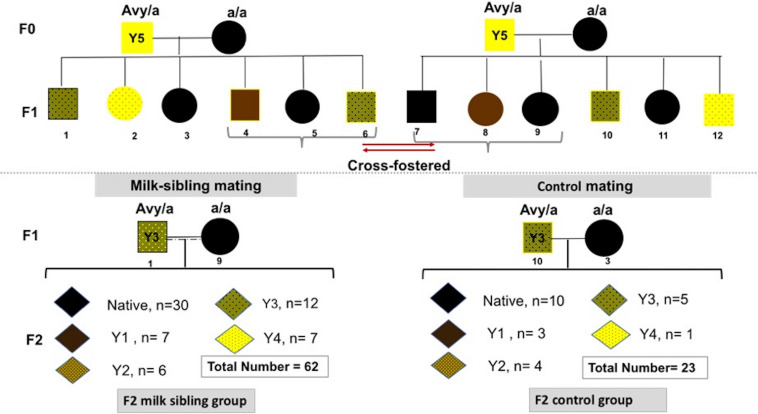
Experimental scheme demonstrating cross-fostering. A*^*vy*^*/a male and a/a female mice (F0) were mated. The numbers under the puppies are given as a representation to explain the model. Three offspring (F1) from each mother were randomly removed and transferred to the other dam at postnatal day 1. Beginning at the postnatal 6th week, F1 mice were mated with their cross-siblings or non-consanguineous mice to obtain F2 milk-sibling or F2 control groups. Starting from the F0 generation, the cross-fostering model was repeated 3 times and recurrent matings of certain animals in the F1 generation were performed to produce F2 control and F2 milk-sibling groups. Distribution of F2 offspring coat color in milk-sibling and control groups after repeated matings is given.

Heterozygous viable yellow males (*A*^*vy*^/*a*) were paired with non-mutant females (a/a; generation F0). Each mother had 6-9 pups (F1 generation) with a continuum of coat colors from all brown (Y0) to all yellow (Y5) with transitional mottled patterns (Y1–Y4). Cross fostering was performed by removing pups from one dam and transferring them to another lactating dam with pups of the same approximate age (Lohmiller and Swing, 2006). To establish a successful cross-fostering model and reduce the stress of dams and pups, previous published guidelines were followed (Fahrenkrog et al., 2004; Lohmiller and Swing, 2006). Three offspring (F1) from each mother were randomly selected, marked with intradermal staining, and transferred to the other dam at postnatal day (PND)1 (Figure 1). Pups were reared in these nests until weaning on PND28. At the end of the fourth week, the offspring were separated from the mother and started to be fed with a standard mice diet (Bilyem standard mouse pellet, Ankara, Turkey: 23% crude protein, 8% ash, 7% crude cellulose, 5% crude fat, and 2,900 kcal/kg). Beginning at the postnatal 6th week, F1 mice were mated with their milk-siblings or unrelated mice to obtain the F2 milk-sibling group and F2 control group. Starting from the F0 generation, the cross-fostering model was repeated 3 times. Proper animals in the F1 generation were mated repeatedly to produce F2 control and F2 milk-sibling groups. Finally, a colony composed of 122 animals (F1 and F2 generation) was yielded.

### Experimental Procedures in the Offspring

Since miRNAs in breast milk and tissues can vary in quantity and quality during different periods of breastfeeding and development, breast milk from mothers and tissues from developing puppies were obtained on PN10 to provide standardization. On PND10, when coat colors became clear, all of the animals were photographed and coat colors were classified from Y0 to Y5 by two researchers (Cooney et al., 2002). After coat color determination on PND10, 25 pups in the F2 milk-sibling group and 9 pups in the F2 control group were sacrificed.

Liver and gonad tissues of sacrificed animals were placed in liquid nitrogen in a protective container and stored at −80°C for real-time quantitative polymerase-chain-reaction (qPCR) studies. The remaining animals (37 pups in the F2 milk-sibling group and 14 pups in the F2 control group) were followed long term for phenotypic changes.

### Follow Up of Phenotypic Characteristics

At follow-up, biweekly body weight measures, fasting blood glucose levels at 5th and 8th month, and changes in the animals’ appearance or behavior were monitored closely. Whole blood glucose level was measured using a hand-held whole-blood glucose monitor (FreeStyle Optium Neo Blood Glucose and Ketone Monitoring System, sponsored by Abbott Diabetes Care) after 6 h of fasting period. Blood samples (5 μl or less) were taken from tail tip after needle punctuation. Sex and species specific reference ranges were used to define abnormal levels (Ayala et al., 2010; Benede-Ubieto et al., 2020). Lifespan of each animal were recorded individually. The autopsy of the animals resulting in spontaneous mortality was performed and macroscopically observed pathological changes were recorded. Additionally, three animals from each group were sacrificed at about 18 months and liver tissues evaluated by histological methods.

### RNA Isolation

Total RNA was isolated from breast milk (F0 and F1 generations) and liver and gonad tissues (F2 generation) using TRIZOL (Roche, Germany; Trakunram et al., 2019). Concentration and purity of RNA for each sample were confirmed using a Nanodrop1000 (Thermo Fisher Scientific, MA, United States).

### miRNA Expression Levels

cDNA was synthesized from the obtained RNA samples using a cDNA synthesis kit (Thermo Fisher Scientific) according to the manufacturer‘s protocol. cDNA samples were quantified using the Biomark Real Time PCR System for expression levels of 384 miRNAs (Tan et al., 2015).

The expression profiles of 384 miRNAs were determined using the following panels: miRNA serum plasma (MIMM-106Z, Qiagene, Germany), Neurological Development and disease (MIMM-107Z, Qiagene), Immunopathology (MIMM-104Z, Qiagene) and Cell Development and Differentiation (MIMM-103Z, Qiagene). ACTB and GAPDH were used as housekeeping genes. All samples were performed in duplicate and the values were normalized using the 2^−Δ*Ct*^ method (Livak and Schmittgen, 2001). All stages of PCR studies were performed at Erciyes University’s Betul-Ziya Eren Genome and Stem Cell Center (GENKOK).

### Reverse Transcription and Pre-amplification Reactions

Isolated RNA samples were reverse-transcribed into cDNA in 5 μl final reaction volumes using the TaqMan MicroRNA Reverse Transcription Kit (P/N: 4366596; Life Technologies, Foster City, CA, United States). All reactions were performed as specified in the manufacturer’s protocol: 2 μl total RNA were added to 3 μl RT reaction mix (Megaplex RT Primers 10X dNTPs with 100 mM dTTP, 50 U/μl MultiScribe Reverse Transcriptase, 10X RT Buffer, 25 mM MgCl_2_, 20 U/μl RNase Inhibitor, and nuclease free water). Reverse transcription was performed using a SensoQuest GmbH Thermal Cycler (Göttingen, Germany). Reaction conditions were 16°C for 120 s, 42°C for 60 s, and 50°C for 1 s repeated for 40 cycles. The final step was 85°C for 300 s and 4°C for at least 600 s until further processing or storage. cDNA samples were stored at −80°C until PCR analysis. Pre-amplification was performed following reverse transcription using the TaqMan SybrMaster Mix (P/N 4309155; Life Technologies). All reactions were performed as specified in the protocols of the manufacturer. For pre-amplification, 2 μl 1:5 diluted RT product was added to 3 μl PreAmp mix. miRNA TaqManPreAmp Thermal Protocol was performed using a SensoQuest GmbH Thermal Cycler (Göttingen) as follows: 95°C for 600 s, 55°C for 120 s, and 72°C for 120 s followed by 18 cycles of 95°C for 15 s, 60°C for 240 s, and 600 s at 99.9°C followed by a rest period at 4°C (Peng et al., 2019).

### qPCR

Quantitative Real-Time PCR reactions (qPCR) were performed using the high-throughput BioMark Real-Time PCR system (Fluidigm, South San Francisco, CA, United States). Pre-amplified cDNA samples were diluted with low EDTA (0.1 mM) TE Buffer (1:5). TaqManSybr Master Mix (490 μl; P/N 4309155; Life Technologies) and GE Sample Loading Reagent (49 μl; P/N 85000746; Fluidigm, South San Francisco, CA, United States) were mixed and 3.85 μl was pipetted into a 96 well plate with 3.15 μl 1:10 diluted pre-amplified cDNA into each well and mixed. Then, 5 μl of this mixture and 4 μl 1:1 diluted assay mixture were pipetted into assay inlets of a 96.96 Dynamic Arrays (Fluidigm). The BioMark IFC controller HX (Fluidigm) was used to distribute the assay and sample mixes from the loading inlets into the 96.96 Dynamic array reaction chambers for qPCR by Fluidigm’s Integrated Fluidic Circuit Technology. qPCR steps were performed using the BioMark System according to the following protocol: 50°C for 120 s, 70°C for 1.800 s, and 25°C for 600 s. Then UNG and Hot start protocol were performed at 50°C for 120 s and initial denaturation at 95°C for 600 s. Finally, PCR cycles followed with 30 cycles at 95°C for 15 s (denaturation) and 60°C for 60 s (annealing) (Kocyigit et al., 2017). We used a combination of two reliable housekeeping genes’ [GAPDH (coding for glyceraldehyde 3-phospate dehydrogenase) and Beta-actin] expression for internal normalization after a preliminary test of the relative expression variance of these two internal controls across different sample types (Tan et al., 2015).

### *In silico* Identification of miRNA Target Genes and Related Pathways

To identify potential target genes of the significantly differentially expressed miRNAs, we conducted an *in silico* analysis using DIANA Tools with mirPath v2.0, microT-CDS v5.0^1^ that support all analyses for KEGG (Kyoto Encyclopedia of Genes and genomes, https://www.genome.jp/kegg/) molecular pathways, as well as multiple slices of Gene Ontology (GO, http://geneontology.org/) in Mus musculus (Kanehisa and Goto, 2000; Vlachos et al., 2012; Kanehisa et al., 2016). Analysis using DIANA were performed with the default parameters (*p* value threshold: 0.05, microT threshold: 0.8) (Vlachos et al., 2012). The web server identifies miRNAs targeting the selected pathway and ranks them according to their enrichment *p* values. We implemented functional enrichment analysis of miRNA target genes using annotation from the KEGG Pathway Database (Kanehisa and Goto, 2000; Kanehisa et al., 2016). Additionally, to functional annotation of miRNAs and miRNA combinations DIANA and KEGG as well as GO were used using all datasets or their subsets (genes union and pathways union parameters were selected). The biology process terms with *p <* 0.05 were considered statistically significant. Targeted Pathways clusters/heatmaps were generated from DIANA. By selecting Targeted Pathways Clusters/heatmap, miRpath flags all the significant pathways (with *p* values < 0.05) with 0 and the other pathways with 1. The miRD^2^, microRNA.org-Targets and Expression, and miRbase: the microRNA database were also used to predicted miRNA-target interactions (http://www.microrna.org/microrna/home.do; http://mirbase.org/; http://mirdb.org/). Ensembl and miRbase were used to support miRNA nomenclature history (http://mirbase.org/; http://www.ensembl.org/index.html). The combination of validated and predicted miRNA-target interactions were used for further analyses with qPCR.

### Determination of Gene Expression Levels

Gsk3b, Mapk8, and Ppargc1a genes were selected among miRNA target genes and expression levels were determined in all samples. To determine the expression levels of these genes, cDNA was synthesized from the obtained RNA samples using a cDNA synthesis kit (Thermo Fisher Scientific). cDNA procedure was conducted according to the manufacturer‘s protocol. cDNA samples were quantified using the Roche 480 Real Time PCR System for expression levels of Gsk3b, Mapk8, and Ppargc1a genes (Kocyigit et al., 2017).

Gsk3b, Mapk8, and Ppargc1a expressions represented in Figures 7, 8 were normalized to Beta-Actin and GAPDH genes using the 2^–Δ*Ct*^ method (Livak and Schmittgen, 2001). Expression changes in the F2 cross-fostering group compared to the F2 control group were calculated and shown as fold change in the graphs.

### Histological Analysis

Liver tissues of three subjects from each group around the 18th month of age were fixed in formalin and processed. Formalin-fixed paraffin-embedded tissue sections were stained with hematoxylin and eosin for light microscopy. The sections were also stained with Prussian Blue and Masson trichrome to demonstrate tissue iron and fibrosis, respectively. A semi-quantitative scoring system was used to grade steatosis, hepatic nuclear degeneration, fibrosis, lymphoid aggregates and hemosiderin pigmentation (0 normal 1 mild, 2 moderate, and 3 severe histopathological changes) (Goodman, 2007; Liang et al., 2014).

### Statistical Analysis

Data were collected using Fluidigm Real-Time PCR Analysis Software, the linear derivative baseline correction method, and the auto global Cq threshold method. System-given Cq values of 999 and values larger than 26 were considered non-speci?c and beyond detection limits and removed. Median limit of detection Cq values were calculated across all arrays to assign missing values. Data normalization was performed using the 2^–Δ^ ^Δ^ ^*Ct*^ method (Livak and Schmittgen, 2001). All of the analysis was performed through a web based software^3^.

MicroRNAs fold change values were calculated using R 3.2.2 software (limma) and easy ROC packages (R Core Team, 2015). A *p* value < 0.05 was considered statistically significant. Benjamini-Hochberg error test was used to adjust *p* values by taking into account multiple tests (Vlachos and Hatzigeorgiou, 2017). The Student’s *t*-test was used for comparisons between groups. A *p* value < 0.05 was considered statistically significant.

SPSS IBM 24 statistical package program was used for comparing phenotypic features of the F2 control and F2 milk-sibling groups. The normal distribution of the data was evaluated using a histogram, q-q graphs, and the Shapiro–Wilk test. Categorical data were evaluated using the chi-square test. Homogeneity of the variances tested by Levene’s test. A resulting *p*-value > 0.05 means that variances are equal and then further parametric tests are suitable. If a resulting *p* value under 0.05, Mann–Whitney *U* test was performed.

## Results

**FIGURE 2:**
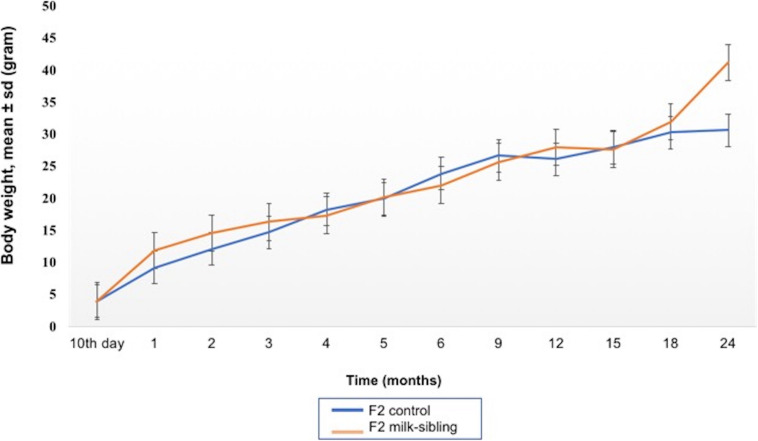
Mean body weight follow up of the two groups. The difference between the groups at the 1st, 2nd, and 24th months were statistically significant (*p* values: 0.014, 0.029, and 0.044, respectively).

**FIGURE 3:**
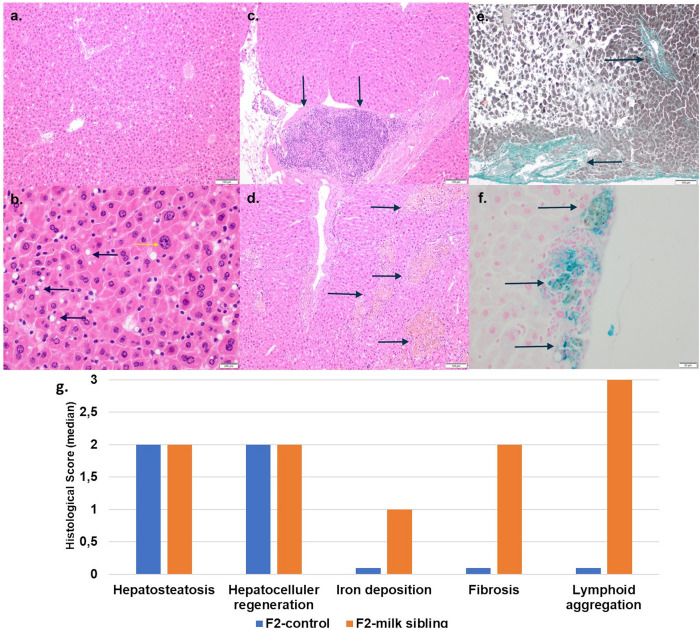
Liver tissue histology. **(a)** F2 Control group: mild liver steatosis (H&E, x10 magnification). **(b)** F2 Control group: Mild liver steatosis (black arrows), hepatocytic regenerative changes, nucleomegaly, and coarse chromatin (yellow arrows; H&E, x10 magnification). **(c)** F2 Milk-sibling group: lymphoid aggregates (black arrows; H& E, x10 magnification) Focal. **(d)** F2 Milk-sibling group: Yellowish pigmented foci (black arrows; H& E, x10 magnification). **(e)** F2 Milk-sibling group: fibrosis around central ven (black arrows; Masson trichrome, x20 magnification). **(f)** F2 milk-sibling group: Hemosiderin containing nodules (black arrows; Prussian blue staining, x20 magnification). **(g)** Comparison of median liver histology scores across the F2-control and F2 milk-sibling groups sibling (statistical analysis could not be performed because of the small sample size).

Coat color distribution was not significantly different between the two groups. Initial mean body weights were similar in both groups. However, the F2 milk-sibling group showed higher weight gain during the first two postnatal months than the F2 control group (*p* < 0.05). Although this difference was not significant during the intervening months, by the 24th month, the mean body weight was significantly higher in the F2 milk-sibling group (*p* < 0.05). Mean fasting blood glucose levels at the 5th and 8th months were not significantly different between the two groups (Table 1 and Figure 2). In the liver histological evaluation, the control group (having Y0, Y2, and Y3 coat colors) showed non-specific changes except for mild steatosis and hepatocytic regenerative changes. These non-specific changes were also seen in the F2 milk-sibling group (Y0, Y2, and Y4 coat colors). However, they also showed some additional pathological changes such as hemosiderin depositions, lymphoid aggregates, and fibrosis (Figure 3).

**TABLE 1:** Phenotypic characteristics of the F2 generation.

	F2-cross-fostering *n* = 62	F2-control *n* = 23	*P*
Sex, female, n (%)	31 (50.0)	15 (65.2)	0.266*
Coat color			
Y0 (Native)	30 (48.3)	10 (43.5)	0.058*
Y1	7 (11.2)	3 (13.0)	
Y2	6 (9.7)	4 (17.4)	
Y3	12 (19.4)	5 (21.7)	
Y4	7 (11.2)	1 (4.3)	
Y5	0	0	
Long term follow up	*n* = 37	*n* = 14	
Blood glucose mg/dl (mean ± sd)			
5th month	110.5 ± 13.9	94.0 ± 23.2	0.062**
8th month	100.5 ± 13.9	90.0 ± 23.2	0.101**
Mean lifespan, days (mean ± sd)	387.9 ± 234.9	589.7 ± 208	0.011**

*Chi square test. **Student *t* test.

**FIGURE 4:**
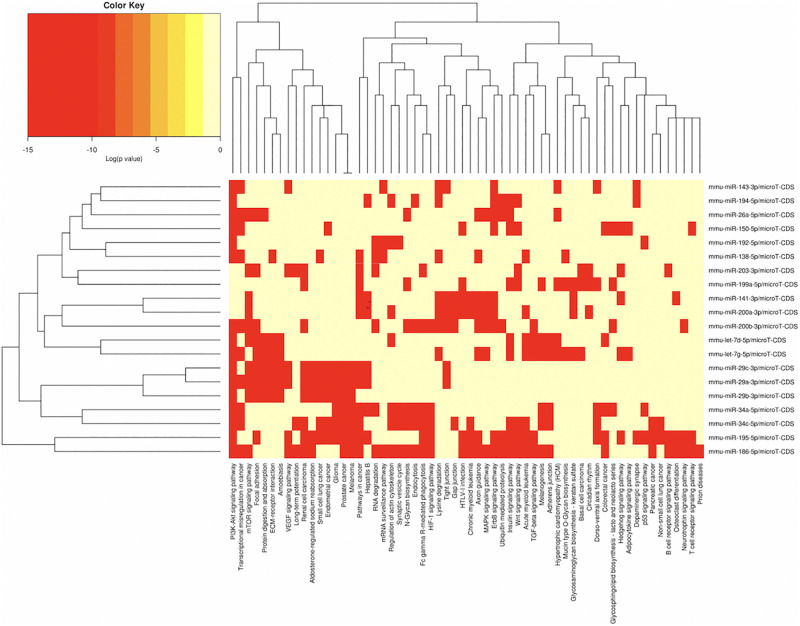
miRNAs vs pathways heatmaps generated from DIANA miRPath. Targeted pathway heatmap shows differentially expressed miRNAs across F2 ovaries from milk-siblings and control groups. Darker colors represent lower significance values. Heatmaps on both axes represent hierarchical clustering results for miRNAs and Pathways, respectively. miRNA axis shows miRNAs clustered together by similar pathway targeting patterns. Similarly, an analogous clustering is shown on the pathway axis.

**FIGURE 5:**
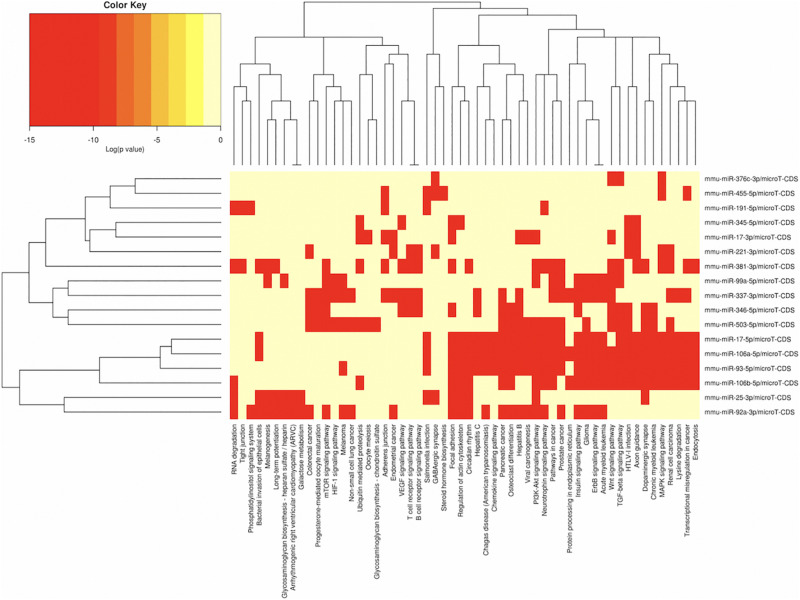
miRNAs vs pathways heatmaps generated from DIANA miRPath. Targeted pathway heatmap shows differentially expressed miRNAs across F2 testes from milk-siblings and control groups. Darker colors represent lower significance values. Heatmaps on both axes represent hierarchical clustering results for miRNAs and Pathways, respectively. miRNA axis shows miRNAs clustered together by similar pathway targeting patterns. Similarly, an analogous clustering is shown on the pathway axis.

**FIGURE 6:**
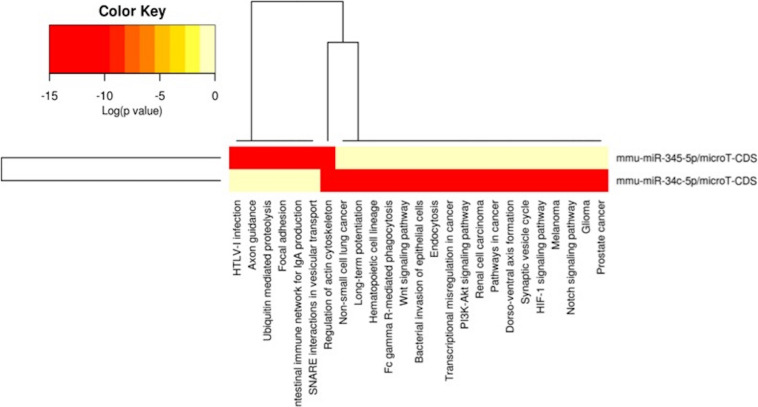
miRNAs vs pathways heatmaps generated from DIANA miRPath. Targeted pathway heatmap shows differentially expressed miRNAs across F2 livers from female milk-siblings and control groups. Darker colors represent lower significance values. Heatmaps on both axes represent hierarchical clustering results for miRNAs and Pathways, respectively. miRNA axis shows miRNAs clustered together by similar pathway targeting patterns. Similarly, an analogous clustering is shown on the pathway axis.

In order to understand the epigenetic mechanisms leading to these phenotypic changes, we performed miRNA expression analysis to identify differentially expressed miRNAs across milk-sibling and control groups. Comparisons of miRNA expression patterns were performed between the gonad and liver tissues of the F2 control and the F2 milk-sibling groups. Expression patterns of miRNAs demonstrating significant differences in paired comparisons were further analyzed using web-based bioinformatics tools to determine potential regulated targets and pathways (Vlachos et al., 2012). Based on our data showing that F2 milk-siblings had higher body weight and shorter life expectancy compared to control counterparts (Table 1 and Figure 2), we focused on the signaling pathways regulating lifespan and metabolism (Figures 4–6). Thus, we concentrated on heat maps generated from F2-ovaries (Figure 4), F2-testes (Figure 5), and F2-livers (Figure 6). We observed the PI3K-Akt signaling pathway, ErbB signaling pathway, mTOR signaling pathway, MAPK signaling pathway, transcriptional misregulation in cancer, pathways in cancer, and insulin signaling pathway were most commonly affected in the milk-siblings group in comparison to controls. These signaling pathways are part of nutrient-sensing systems known to regulate reproductive and somatic aging as well as metabolic syndrome and its complications, including fatty liver disease, respiratory disease, diabetes, cardiovascular disease, osteoarticular disease, and cancer (Templeman and Murphy, 2018; Fadini et al., 2011).

**FIGURE 7:**
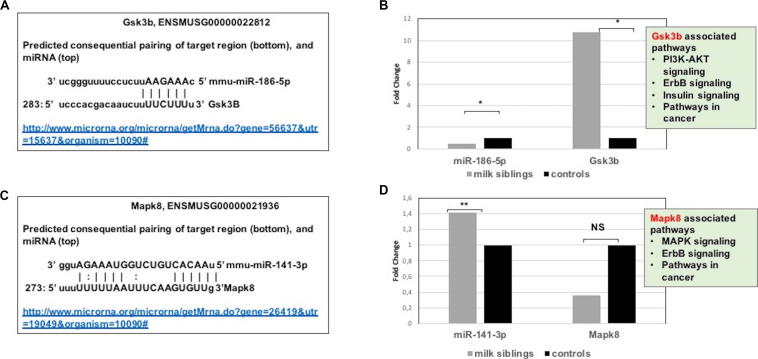
**(A)** Schematic representation describing sequence alignment of mm-miR-186-5 with the 3′-UTR of Gsk3b (http://www.microrna.org/microrna/getMrna.do?gene=56637&utr=15637&organism=10090). **(B)** Graph shows corrected fold changes in miR-186-5p and Gsk3b expression levels (milk-siblings to control groups) in the ovary tissues. ACTH was used as a reference gene. Values from control groups were set at 1. (http://www.microrna.org/microrna/getMrna.do?gene=26419&utr=19049&organism=10090). **(C)** Schematic representation describing sequence alignment of mm-miR-141-3p with the 3′-UTR of Mapk8. **(D)** Graph shows corrected fold changes in mm-miR-141-3p and Mapk8 expression levels (milk-siblings to control groups). ACTH was used as a reference gene. Values from control groups were set at 1. *P < 0.05, **P < 0.01.

**FIGURE 8:**
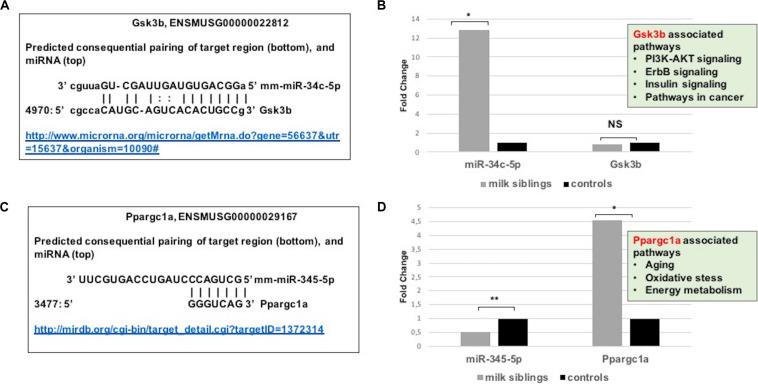
**(A)** Schematic representation describing sequence alignment of mm-miR-34c-5p with the 3′-UTR of Gsk3b (http://www.microrna.org/microrna/getMrna.do?gene=56637&utr=15637&organism=10090). **(B)** Graph shows corrected fold changes in mmu-miR-34c-5p and Gsk3b expression levels (milk-siblings to control groups) in the liver tissues. ACTH was used as a reference gene. Values from control groups were set at 1 (http://mirdb.org/cgi-bin/target_detail.cgi?targetID~=~1372314). **(C)** Schematic representation describing sequence alignment of mm-miR-345-5p with the 3’-UTR of Ppargc1a. **(D)** Graph shows corrected fold changes in mm-miR-345-5p and Ppargc1a expression levels (milk-siblings to control groups). ACTH was used as a reference gene. Values from control groups were set at 1. *P < 0.05, **P < 0.01.

Targeted pathway heatmaps show that miR-186-5p targets most of the signaling pathways playing a role in survival and metabolism. Target prediction analysis showed that miR-186-5p potentially targets Gsk3b (Figure 7A), which is involved in many pathways such as the PI3K-akt signaling pathway, ErbB signaling pathway, pathways in cancer, and insulin signaling pathway (Figure 4A). Gsk3b expression in F2-ovaries was analyzed using RT-qPCR to examine if there is a link between Gsk3b and miR-186-5p expression. miR-186-5p expression decreased in F2 milk-siblings, whereas Gsk3b increased compared to control counterparts (Figure 7B).

Following the same logic, we found that miR-141-3p targets most of the survival and metabolism related pathways, including the MAPK signaling pathway, mTOR signaling pathway, ErbB signaling pathway, and pathways in cancer (Figure 4). Target prediction analysis showed that miR-141-3p potentially targets Mapk8 (Figure 7C), which is involved in the MAPK signaling pathway, ErbB signaling pathway, and pathways in cancer When we analyzed Mapk8 expression in F2-ovaries using RT-qPCR, we observed that miR-141-3p expression increased in F2 milk-siblings, whereas Mapk8 expression decreased compared to the control group (Figure 7D).

Because metabolic homeostasis is dependent on proper liver function, we evaluated miRNAs and their potential targets’ expressions in liver tissues. We detected only two miRNAs differentially expressed in milk-siblings in comparison to control groups in liver (miR-345-5p and miR-34c-5p; Figure 6). These miRNAs target the PI3K-Akt signaling pathway, transcriptional misregulation in cancer, pathways in cancer, and mTOR signaling pathway, which are among the target pathways significantly affected in F2-gonad tissues. We investigated potential targets of mmu-miR-34c-5p and mmu-miR-345-5p using web-based bioinformatics tools (Figure 8). We identified a complementary site for mmu-miR-34c-5p in the 3’UTR of Gsk3b mRNA (Figure 8A). miRNA expression profiling revealed that mmu-miR-34c-5p expression increased in milk-siblings, whereas miR-345-5p expression decreased in milk-siblings in comparison to their control counterparts (Figures 8B,D). Expectedly, RT-qPCR analysis of Gsk3b, a predicted target of mmu-miR-34c-5p, decreased in milk-siblings and Ppargc1a, a predicted target for mmu-miR-345-5, increased in milk-siblings compared to their control counterparts (Figures 8B,D; for entire list and *p* values, see Supplementary Data).

## Discussion

The most striking result of study was that mating of milk-siblings resulted with various pathologies in offspring possibly because of the shared breast milk that can affect epigenetic regulation mechanisms. Results revealed that the life expectancy of the offspring obtained from milk-siblings mating was much shorter than the offspring from control matings. Offspring of milk-siblings were more obese during the aging period and histopathological examination of liver tissues revealed abnormal findings that were not shown in the offspring from control matings such as lymphoproliferative nodules, abnormal iron accumulation, and fibrosis.

In our study, we performed miRNA microarray to profile mouse miRNAs in ovaries, testes, and livers and make expressional comparison between milk-siblings and control groups. The number of significantly differentially expressed miRNAs was higher in ovaries than testes than livers. Interestingly, these differentially expressed miRNAs mostly targets metabolism, survival and cancer associated pathways with very small *p*-values. Notably, these pathways strongly associated with the phenotypes we observed. Particularly, mTOR signaling, AMPK signaling, FOXO signaling, insulin signaling that are commonly targeted by miRNAs differentially expressed in milk-siblings are nutrient-sensing systems determine reproductive status and somatic tissue maintenance with age (Fadini et al., 2011; Templeman and Murphy, 2018). Accumulating evidences highlights the fact that longevity and metabolic signals interplay in a complex way in which lifespan appears to be strictly dependent on substrate and energy bioavailability. These pathways also determine the development of the metabolic syndrome and its complications affecting disparate organs and systems, such as fatty liver disease, respiratory disease, diabetes, cardiovascular disease, osteoarticular disease, and cancer (Fadini et al., 2011; Templeman and Murphy, 2018). Thus, it is not surprising that differentially expressed miRNAs mostly clustered into these pathways. For instance, in our study we found that while miR-186-5p downregulated in milk-siblings Gsk3b upregulated in milk-siblings compared to control groups. Downregulation of miR-186-5p and upregulation of GSK3B have been reported in ovarian carcinoma (Hilliard et al., 2011; Templeman and Murphy, 2018). Overexpression of GSK3B has been implicated in insulin resistance, polycystic ovary syndrome (PCOS), platinum-resistance in ovarian cancer (Cai et al., 2007; Goodarzi et al., 2007; Hilliard et al., 2011). In addition, we detected upregulation of miR-141-3p in milk-siblings and downregulation of its potential target MAPK8 in milk-siblings. Interestingly, miR-141-3p upregulation and MAPK8 (JNK1) signaling downregulation have been reported in platinum-based chemotherapy in ovarian cancer cell lines (Ying et al., 2015; Li et al., 2018). MAPK8 has been also implicated in the aging-related disease and overexpression of JNK1 in roundworms was reported to increase lifespan (Oh et al., 2005). Overall, even though our bioinformatics and gene expression studies support the data gathered from phenotypic observations, it is necessary to perform further analysis to conclude that these miRNAs and their potential targets specifically play roles in. We have not analyzed animals for insulin-resistance, PCOS or ovarian cancer cells; it would be worth studying the potential role of miR-186-5p/Gsk3b axis in ovarian tissues. Considering the fact that liver plays an essential role in metabolic homeostasis, we evaluated miRNAs and their potential targets expressions in liver tissues. We detected only two miRNAs statistically differentially expressed in milk siblings in comparison to control groups. miR-34c-5p showed a higher level in milk-siblings and accordingly its potential target Gsk3b was lower in the same tissues compared to control counterparts. It was shown that miR-34c-5p upregulated in hepatitis B-related acute-on-chronic liver failure (Ding et al., 2015). In addition, increased expression of miR-34c-5p was shown in hypoxic rat liver (Zhi et al., 2017). Accordingly, Gu et al. (2018) showed that Gsk3b signaling decreased under intermittent hypoxic conditions. These studies imply that increased expression of miR-34c-5p and decreased activity of Gsk3b signaling might be a response to cellular stress conditions. Furthermore, we found a decreased expression of miR-345-5p and an increase expression of its potential target gene Ppargc1a in milk-siblings. Interestingly, Chen et al., performed an integrated analysis of miRNA and gene expression profiles highlighted a functional miRNA-gene regulatory module associated with liver fibrosis and listed miR-345-5p among decreased miRNAs (Chen et al., 2017). Similarly, histological analysis showed fibrosis in milk-siblings’ livers that have decreased expression of miR-345-5p. In addition, we observed fat accumulation in livers of milk-siblings. Liver fibrosis (Stal, 2015) and impaired PPARGC1A activity are associated with non-alcoholic fatty liver diseases (Aharoni-Simon et al., 2011). Taken together our results supports that miRNAs likely mediate the phenotypes observed in survival-, obesity- related, transgenerational metabolic disturbances.

There might be a period in which offspring are susceptible to breast milk induced epigenetic changes. The period that begins with conception and covers the first 2 years of life is suggested as the most active period in terms of epigenetic regulation, especially in terms of DNA imprinting. Therefore, this period is referred to as “1000 day period” (Shenderov and Midtvedt, 2014; Walker, 2016; Indrio et al., 2017; Linner and Almgren, 2020). Growing amount of evidence supports that epigenetic programming affected by early nutrition may result in adult disease in the long run (Shenderov and Midtvedt, 2014; Indrio et al., 2017). Considering that, there may be a sensitivity period to epigenetic factors in breast milk all suckling pups were weaned on the same PND in this study.

Epigenetic developmental plasticity allows an organism to adapt to environmental signals, especially during fetal and early life (Shenderov and Midtvedt, 2014). The cross fostering may have disrupted the epigenetic developmental plasticity starting during fetal life and continuing during the early postnatal life. Furthermore, the milk of each mother is specific to her own baby, and the maternal-fetal interaction during fetal life may affect the composition of breastfeeding (Twigger et al., 2015) and the baby’s tolerance to the biological mothers’ milk. Therefore, epigenetic differences in F2 milk-sibling group may have been aroused not only due to mating of milk-siblings, but also due to the disruption of epigenetic developmental plasticity due to lack of breast milk of their own and breastfeeding by foster-mother.

Since the genetic structures and environmental conditions of the cross-sibling and control groups were the same in this model, breast milk was supposed to be the only responsible factor for existing epigenetic changes. Because of the similarity of the genetic backgrounds and environmental exposures of the two groups, posttranscriptional epigenetic mechanisms, especially through miRNAs, were investigated instead of DNA methylation patterns or histone modifications. Small transcoded RNAs (microRNA, pi-RNA, etc.) play an important role in transgenerational epigenetic inheritance. Sperm not only transfers DNA to ovum, but also transfers different kinds of RNAs including miRNAs and pi-RNAs. These transferred RNAs play an important role in embryo development by influencing various mRNA expressions (Smythies et al., 2014). Future experiments analyzing the milk in each generation could be performed in order to establish the precise participation of miRNAs.

When the results of this study are interpreted, the following conclusions may be drawn: i.e., substances that may affect epigenetic regulation mechanisms may be transferred to F1 generation via breastfeeding, ii. epigenetic interactions or epimutations occurring in F1 generation can be transferred to the next generation. It is not clear whether this kind of inheritance occurs in monoepigenic or polyepigenetic as in the other genetic diseases. The results of this study can not claim exactly the mechanisms by which breast milk affects meiotic epigenetic inheritance and it is not exactly known yet, how it has been carried over generations. If the results of this study are supported by further studies, it may be questionable that milk-sibling marriages cause hereditary diseases as in the consanguineous marriages.

There are several limitations of this study. Although bioinformatics approaches were used to predict functionality of miRNAs, experimental validation is necessary to show miRNA: gene interactions. Secondly, in the F1 generation, cross fostering may have stressed animals and may have affected subsequent metabolic responses in the F2 generation. However, to test the effect of milk-siblingship on subsequent generations, this breeding scheme allowed us to keep the genetic background of the animals comparable in the two F2 groups. Furthermore, consequences of disruption of maternal-fetal dyads by cross fostering should also be considered when interpreting the results as discussed above. Small sample size was another important limitation of the study. Further experiments are needed to the differentiate the epigenetic effects of milk-sibling and cross fostering on future generations.

## Conclusion

The results of this study support that epigenetic regulation mechanisms may be transmitted to the baby through breast milk and these epigenetic interactions or epimutations may be transferred to the next generation transgenerationally. The current results indicate that milk-sibling mating may cause various diseases in offspring. Results may also indicate the heritable epigenetic effects of cross-fostering on future generations. Similar results are not verified in humans. Nevertheless, with these remarkable findings, it is time to reconsider what we know about this issue.

## Code Availability

DIANA Tools with mirPath v2.0, microT-CDS v5.0 (see text footnote^1^), KEGG (Kyoto Encyclopedia of Genes and genomes, https://www.genome.jp/kegg/) molecular pathways, and multiple slices of Gene Ontology (GO, http://geneontology.org/) in Mus musculus were used for bioinformatics analysis (Kanehisa and Goto, 2000; Vlachos et al., 2012; Kanehisa et al., 2016).

## Data Availability Statement

The datasets presented in this study can be found in online repositories. The names of the repository/repositories and accession number(s) can be found in the article/Supplementary Material.

## Ethics Statement

The animal study was reviewed and approved by Dokuz Eylul University Multidisciplinary Animal Laboratory Ethical Committee.

## Author Contributions

HO revealed the main hypothesis. HO, FT, and YÖ designed the study. FT and PA performed most of the experiments. ST performed the PCR studies and wrote some part of the manuscript. PK performed the data and bioinformatics analysis and also contributed to writing. EÖ performed the histological analysis. ET contributed to PCR studies. OY, ND, and AK contributed to the experimental assistance. YÖ provided conceptual advice. FT and PK wrote the manuscript with comments from all authors. All authors contributed to the article and approved the submitted version.

## Conflict of Interest

The authors declare that the research was conducted in the absence of any commercial or financial relationships that could be construed as a potential conflict of interest.

## Footnotes

1 http://diana.imis.athena-innovation.gr/DianaTools/index.php?r=site/index

2 http://mirdb.org/

3 http://www.qiagen.com/us/shop/genes-and-pathways/data-analysis-center-overview-page/

## Supplementary Material

Supplementary Additional File 1 Comparison of F2 female gonads (milk sibling vs control).

Supplementary Additional File 2 Comparison of F2 livers (milk sibling vs control). Comparison of F2 livers.

Supplementary Additional File 3 Comparison of F2 male gonad (milk sibling vs control).

Supplementary Additional File 4 miRNA expression-Comparison of F2 female gonads (milk sibling vs control).

Supplementary Additional File 5 miRNA expression comparison of F2 male gonads (milk sibling vs control).

Supplementary Additional File 6 miRNA expression Comparison of F2 livers (milk sibling vs control).

Supplementary Additional File 7 RT-mRNA expression a. Comparison of F2 female gonads (milk sibling vs control) b. Comparison of F2 livers (milk sibling vs control).

Supplementary Additional File 8 Representation of the biological process that Mapk8 involved in. The data are obtained from Ensembl (http://www.ensembl.org/index.html).

Supplementary Additional File 9 Representation of the biological process that and Ppargc1a involved in. The data are obtained from Ensembl (http://www.ensembl.org/index.html).

Supplementary Additional File 10 Representation of the biological process that Gsk3b involved in. The data are obtained from Ensembl (http://www.ensembl.org/index.html).
